# Effect of Copper-Catalyzed Oxidation on the Aggregation of the Islet Amyloid Polypeptide

**DOI:** 10.3390/antiox14111269

**Published:** 2025-10-22

**Authors:** Océane Amilca, Phuong Trang Nguyen, Lucie Perquis, Fabrice Collin, Steve Bourgault

**Affiliations:** 1 Department of Chemistry, Université du Québec à Montréal, Montreal, QC H3C 3P8, Canada; oceane.amilca@utoulouse.fr (O.A.); nguyen.vo_thanh_phuong@uqam.ca (P.T.N.); 2 Quebec Network for Research on Protein Function, Engineering and Applications (PROTEO), Montreal, QC H3C 3P8, Canada; 3 Laboratoire Softmat, Université de Toulouse, CNRS UMR 5623, 31400 Toulouse, France; lucie.perquis@univ-tlse3.fr

**Keywords:** IAPP, copper, oxidation, amyloid fibrils, aggregation

## Abstract

The islet amyloid polypeptide (IAPP) is a 37-residue peptide hormone secreted by pancreatic β-cells that is known to aggregate into amyloid fibrils. These fibrils accumulate in the pancreatic islets of individuals afflicted with type 2 diabetes and are implicated in β-cell dysfunction. Metal ions such as copper and zinc are known to modulate IAPP fibrillization, yet the role of metal-induced oxidative modifications in this process remains largely unexplored. This study examines the non-enzymatic post-translational oxidation of IAPP and its effects on aggregation using the biologically relevant Cu/O_2_/ascorbate system. Mass spectrometry identified residues within the amyloidogenic region (residues 20–29) as the primary targets of oxidation. These oxidative modifications impaired the formation of cross-β-sheet amyloid fibrils and promoted the accumulation of amorphous aggregates. The H18A IAPP derivative, lacking the key metal-binding histidine, was also examined to assess the impact of sequence variation on oxidation and aggregation. Copper-mediated oxidation of H18A resulted in a broader distribution of oxidation sites and impacts fibril formation. These findings provide preliminary mechanistic insights into copper-induced oxidation and its impact on IAPP aggregation pathways.

## 1. Introduction

Type 2 diabetes (T2D) represents a major global public health crisis, ranking as the eighth leading cause of mortality and disability worldwide [1]. It is primarily characterized by chronic hyperglycemia driven by insulin resistance and progressive dysfunction of the pancreatic β-cells responsible for insulin biosynthesis. Moreover, the presence of amyloid plaques within the islets of Langerhans in the pancreas, observed in over 90% of patients, constitutes a prominent pathological hallmark of T2D [2,3]. These deposits are primarily composed of the islet amyloid polypeptide (IAPP), a 37-residue peptide hormone co-secreted with insulin by pancreatic β-cells. IAPP adopts primarily a disordered conformation under physiological conditions and plays key roles in glucose homeostasis, regulation of gastric emptying, and satiety [4]. Under pathological conditions, increased insulin demand leads to elevated local concentrations of IAPP, likely promoting its aggregation into amyloid fibrils in the vicinity of β-cells [5]. Albeit amyloid deposits can generate local inflammation, most experimental evidence indicates that small, soluble oligomeric intermediates of IAPP, are the most cytotoxic species, being able to disrupt plasma membrane integrity, to trigger β-cell apoptosis and to induce oxidative stress [6,7,8].

While an increased local concentration of IAPP associated with co-secretion of insulin can promote amyloid formation in the Langerhans islets [9,10], other molecular factors could also induce aggregation and tissue deposition. Among these, increased oxidative stress has emerged as a key contributor to T2D pathogenesis, disrupting pancreatic redox balance and exacerbating β-cell dysfunction [11,12,13]. Reactive oxygen species (ROS), including hydrogen peroxide, superoxide anion and hydroxyl radical, are central mediators of oxidative stress. These species cause DNA damage, lipid peroxidation, and induce non-enzymatic post-translational modifications (PTMs) of proteins, ultimately leading to β-cell impairment and apoptosis [14,15,16]. Notably, in T2D, IAPP has been shown to undergo non-enzymatic PTMs in response to oxidative stress. For instance, 4-hydroxynonenal (HNE), a product of lipid peroxidation, forms covalent adducts with histidine residues of IAPP, perturbing its conformation and enhancing its cytotoxicity [17]. A further notable modification involves the formation of advanced glycation end products (AGEs), which have been detected in IAPP and are tightly linked to oxidative stress. Methylglyoxal (MG), a highly reactive glucose-derived metabolite, interacts with residue Lys1 and leads to a glycated form that accelerates amyloid formation [18]. Additionally, AGEs amplify ROS production through the activation of their receptor (RAGE), forming a feedback loop that intensifies oxidative burden [19,20].

In parallel to these oxidative modifications, transition metals, potent modulators of oxidative stress, are also known to modulate polypeptide aggregation and IAPP interactions with polypeptide, as recently exemplified for Zn^2+^ and insulin [21]. Among them, copper has drawn increasing attention for its ability to alter IAPP structure and aggregation kinetics [22,23,24]. Remarkably, elevated levels of copper in the bloodstream have been consistently reported in both T2D patients and diabetic mouse models, suggesting a potential link between metal dysregulation and disease progression [25,26,27,28]. Copper not only impacts the structural dynamics of peptides but could also serve as a redox-active center. In the presence of physiologically reducing agents such as ascorbate, Cu(II) is reduced to Cu(I), which can react with molecular oxygen to produce ROS via redox cycling. This redox cycling has conceptual similarity to Fenton-like mechanism, in which copper reacts with hydrogen peroxide to generate highly reactive hydroxyl radicals (HO^•^) [29]. For example, the amyloid β (Aβ) peptide binds to Cu^2+^ and forms Cu–Aβ complex capable of catalyzing ROS production [30,31]. Moreover, it has been reported that copper binding accelerates aggregation and promotes the formation of toxic species of the Aβ peptide and Tau protein, both polypeptides being implicated in the progression of Alzheimer’s disease [31,32]. Similarly, it was reported that copper binds IAPP, predominantly through the imidazole Nδ1 of His18 residue, thereby inducing significant conformational rearrangements [33]. Upon binding to IAPP monomer, copper modulates the aggregation pathway mainly by interfering with the conformational transitions required for the formation of the organized cross-β-sheet quaternary structure. Notably, it has been shown that this interaction induces the formation of small aggregates [34,35].

Although pancreatic redox stress is well established as a central feature of T2D, and copper is known to both amplify ROS production and modulate the self-assembly of amyloidogenic polypeptides, the impact of metal-catalyzed oxidation on IAPP remains unexplored. In this context, the present study aimed at elucidating the effects of copper-mediated oxidation on IAPP self-assembly using the biologically relevant Cu/O_2_/ascorbate system. Oxidized residues were identified in the amyloidogenic region known to be between residues 20 and 29 [36]. These oxidative modifications significantly impaired the formation of the cross-β-sheet structure, promoting the formation of amorphous aggregates. To gain further insight into the oxidative mechanism and its impact on IAPP self-assembly, the H18A derivative, lacking the key metal-binding imidazole group, was also examined.

## 2. Materials and Methods

### 2.1. Peptide Synthesis, Purification, Characterization and Monomerization

IAPP and its analogs were synthesized by Fmoc solid-phase peptide synthesis on a Rink Amide AM resin, as previously described [37]. Crude peptides were purified by preparative HPLC using a C18 column and a linear gradient of acetonitrile in water containing 0.6% (*v/v*) trifluoroacetic acid (TFA). Fractions were analyzed by high-resolution mass spectrometry using an Agilent 1200 series HPLC coupled to an electrospray time-of-flight mass spectrometer (ESI-TOF) (Agilent Technologies, Palo Alto, CA, USA). Fractions containing the desired peptide with a purity above 95% were pooled and lyophilized. Peptide cyclization was performed by oxidation in 100% dimethyl sulfoxide (DMSO) at room temperature overnight to form the C2–C7 disulfide bridge. The solution was then diluted and subjected to a second round of HPLC purification. The solution was filtered through a 0.22 µm hydrophilic polyvinylidene difluoride (PVDF) membrane and sonicated for 30 min prior to lyophilization. Aliquots of monomerized peptide were prepared by dissolving the lyophilized powder a second time in 100% hexafluoro-2-propanol (HFIP) at a concentration of 1 mg/mL. The solution was then sonicated for 1 h, aliquoted and lyophilized. Monomerized samples were stored in a dried state at −80 °C for a maximum of 4 weeks.

### 2.2. Metal-Catalyzed Oxidation of Peptides and Proteolytic Digestion

Cu(II)-catalyzed oxidation of peptides was performed by incubating the peptide (60 µM) with CuSO_4_ (50 µM) and ascorbate (500 µM) in 50 mM phosphate buffer (pH 7.4) for 30 min at room temperature. These molar ratios were selected according to previous studies using the amyloid β-peptide [30,38,39]. Ammonium bicarbonate buffer (0.1 M, pH 8.0) and α-chymotrypsin (0.05 µg/µL in 0.1% formic acid) were added to obtain a chymotrypsin/peptide ratio of 1:40 (*w/w*). Digestion was performed at 37 °C for 4 h in a Thermomixer (Eppendorf), with mixing for 2 h at 650 rpm. The digestion was quenched by adding 700 µL of water. The resulting solution was purified using a 1 cm^3^ (30 mg) Oasis HLB solid-phase extraction cartridge and eluted with two additions of 500 µL of 100% methanol. Extracts were then evaporated under vacuum and stored at −30 °C until further use.

### 2.3. HPLC-MS/MS Analysis of Peptide Samples

Dried samples were reconstituted in 10% acetonitrile containing 0.2% formic acid. Chromatographic separation was performed using a Shimadzu Nexera UHPLC system (Columbia, MD, USA) equipped with an autosampler maintained at 15 °C. Samples (20 µL) were injected onto an Aeris PEPTIDE XB-C18 column (100 mm × 2.1 mm, 1.7 µm) fitted with a SecurityGuard UHPLC C18-peptide cartridge (Phenomenex, Torrance, CA, USA). Separation was achieved at a flow rate of 300 µL/min using a linear gradient with mobile phase A (0.1% formic acid in water) and mobile phase B (0.1% formic acid in acetonitrile). The gradient program was as follows: 5% B (0–1 min), ramped to 25% B at 10 min, to 50% B at 11 min, then to 85% B at 11 min, and held at 85% B until 14 min. Mass spectrometry analysis was carried out on a high-resolution TripleTOF 5600 Q-TOF instrument (Sciex, Concord, ON, Canada) equipped with a DuoSpray ion source, operated in full-scan positive mode. Ion source parameters were set as: temperature 500 °C, ion spray voltage 5 kV, GS1 and GS2 at 50 psi, and declustering potential at 80 V. Information-dependent acquisition (IDA) was employed for data acquisition over an *m/z* range of 120–1250, with dynamic background subtraction and collision energy of 30 ± 10 V (cycle time: 1.05 s). The top 10 most intense precursor ions were selected for collision-induced dissociation (CID), and MS/MS spectra were acquired over an *m/z* range of 80–1300. Data processing was conducted using the PEAKS software v13 (Bioinformatics Solution Inc., Waterloo, ON, Canada). Oxidized peptides were identified based on specific mass shifts and their absence in control digests. Extracted ion chromatograms (XICs) for native and oxidized chymotryptic peptides were generated using PeakView v2.2 and MasterView v1.1 (Sciex), with a mass tolerance of 0.02 Da to ensure high mass accuracy and confident peptide identification.

### 2.4. Aggregation Kinetics by Thioflavin T Fluorescence

Peptide solutions were prepared by dissolving the lyophilized peptides at a concentration of 50 µM in 50 mM phosphate buffer (pH 7.4). Final peptide concentrations were 12.5 µM and 25 µM and thioflavin T (ThT) concentration was fixed at 40 µM. Aggregation assays were performed at 25 °C without stirring, in sealed, black-wall, clear-bottom 96-well nonbinding surface plates, with a final volume of 100 µL per well. ThT fluorescence was measured every 10 min over a 24-h period using a SpectraMax i3 multimode plate reader (Molecular Devices, San Jose, CA, USA) with excitation at 440 nm and emission at 485 nm. For each experiment, data from triplicate wells were averaged, baseline-corrected by subtracting the corresponding control signal, and plotted as fluorescence intensity in relation to time. The resulting data were fitted to a sigmoidal Boltzmann model given in Equation (1), where Y_0_ and Y_max_ are, respectively, the initial and maximum ThT fluorescence value, t_1/2_ is the time required to reach half of the fluorescence intensity, and k is the elongation rate [40,41].

(1) $$\left(t\right)=Y_{0}+\frac{Y_{max}-Y_{0}}{1+e^{-k\left(t-t_{1/2}\right)}}\;\mathrm{and}\;t_{lag}=t_{1/2}-\frac{2}{k}$$

Lag time and final ThT fluorescence data from at least four independent experiments were averaged and reported as mean ± standard deviation (SD). Statistical analysis was performed with Prism version 6.0 software using Student *t*-test, with a threshold of *p* < 0.05.

### 2.5. Fluorescence Spectroscopy

Peptides were dissolved in 50 mM phosphate buffer (pH 7.4) at a concentration of 60 µM and incubated at room temperature under quiescent conditions for 72 h. ThT and anilino-8-naphthalenesulfonate (ANS) were added to the peptide samples at final concentrations of 40 µM and 60 µM, respectively. Excitation wavelengths were set at 355 nm for ANS and 440 nm for ThT. Emission spectra were recorded from 385 to 585 nm for ANS and from 450 to 550 nm for ThT. Fluorescence measurements were performed in a 10 mm quartz cuvette using a PTI QuantaMaster 400 spectrofluorometer (Edison, NJ, USA). For each measurement, a blank sample containing the fluorophore and buffer was used as a control.

### 2.6. Atomic Force Microscopy

After the desired time of incubation, peptide solutions at a concentration of 60 µM in 50 mM phosphate buffer (pH 7.4) were diluted in 1% acetic acid to reach a concentration of 15 μM and immediately applied on freshly cleaved mica. Acetic acid solution was added to the IAPP samples just prior deposition on the mica to promote adsorption of the peptide aggregates to the negatively charged surface, a procedure not affecting the morphology and stability of the obtained aggregates [42]. The surface was then washed twice with deionized water and air-dried overnight. Samples were analyzed using a Veeco/Bruker Multimode atomic force microscope operated in ScanAsyst-Air mode with a silicon tip (tip radius: 2–12 nm; force constant: 0.4 N/m) mounted on a nitride lever. Images were acquired at a scan rate of 0.9 Hz with a resolution of 1024 scans/min and a scan frame of 10 μm × 10 μm.

## 3. Results and Discussion

### 3.1. Site-Specific Oxidation of IAPP Induced by Copper Redox Cycling

Previous studies have identified His18 and adjacent residues as key sites for Cu(II) binding to IAPP [33,34,43]. Consequently, these residues could also be susceptible to copper-catalyzed oxidation. For the detection of site-specific oxidative modifications, IAPP was incubated with ascorbic acid and copper for 30 min then digested with chymotrypsin prior to analysis by LC-MS/MS. This approach follows the model proposed by Cheignon and colleagues, who investigated the effects of Aβ metal-catalyzed oxidation on ROS production [30]. Table 1 lists the oxidized residues identified, along with their corresponding monoisotopic masses and respective retention times. The digested peptides corresponding to missing cleavages and other modifications are presented in Table S1.

**Figure 1:**
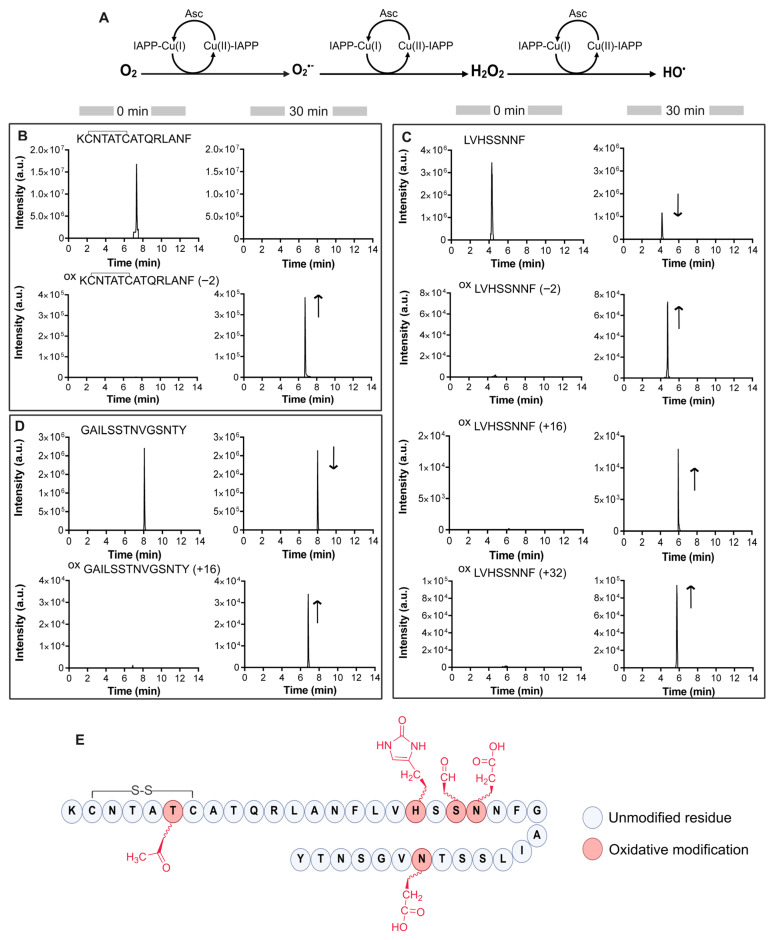
Identification of site-specific IAPP oxidation. (**A**) Generation of ROS catalyzed by copper in the presence of IAPP peptide. Asc refers to ascorbate. (**B**–**D**) Extracted ion chromatograms of chymotryptic IAPP: (**B**) **^1^**KCNTATCATQRLANF**^15^**, (**C**) **^16^**LVHSSNNF**^23^** and (**D**) **^24^**GAILSSTNVGSNTY**^37^** prior and after 30 min oxidation reaction. The direction of the arrows reflects changes in chromatographic peak intensities. (**E**) Summary of Cu-mediated oxidative modifications on IAPP. Oxidative sites identified by MS/MS analysis are highlighted in red.

High-resolution extracted ion chromatograms were acquired with a mass accuracy of ±5 ppm. Representative MS/MS spectra are provided in the Supplementary Information (Figure S1), and a summary of the detected residue modifications is shown in Figure 1E.

Among the detected oxidative modifications, IAPP_(1–15)_ fragment displayed a unique mass shift of −2 Da with a prominent ion detected at *m*/*z* 547.2610 (Figure 1B). This result suggests the loss of two hydrogen atoms, consistent with the dehydrogenation of a threonine side chain, converting the hydroxyl group into a ketone [44].

For the chymotryptic peptide IAPP_(16–23)_, which contains a histidine residue, three different oxidized species were detected (Figure 1C). The first ion, observed at *m/z* 458.2201, corresponds to a −2 Da mass shift, indicative of the oxidation of serine hydroxyl group to 3-oxoalanine, also known as formylglycine [45]. Additional ions detected at *m/z* 467.2253 and 475.2228 exhibit mass shifts of +16 Da and +32 Da, respectively. These products correspond to oxidative modifications, with the +16 Da ion indicative of histidine oxidation to 2-oxo-histidine [46]. The +32 Da shift suggests the incorporation of two oxygen atoms, likely reflecting concurrent oxidation of histidine and asparagine, the latter being converted to aspartate [47]. The relative signal intensities indicate that co-oxidation involving histidine and asparagine, is more prevalent than histidine and serine oxidation alone, as evidenced by peak intensities of approximately 10^5^ versus 10^4^, respectively.

As for the third chymotryptic peptide IAPP_24–37_, LC-MS analysis revealed a single peak at *m/z* 700.3391 corresponding to a +16 Da mass shift. The MS/MS spectra revealed the oxidation of an asparagine residue to aspartate (Figure 1D and Figure S1). Additionally, given that IAPP is naturally amidated at its C-terminus, we investigated the potential deamidation of Tyr37. This modification was observed in both oxidized and non-oxidized forms of the peptide, suggesting that it is not a consequence of ROS-mediated oxidation (Table S1), but rather results from spontaneous hydrolysis occurring during sample preparation [48]. Finally, no oxidative modifications were detected in the control sample containing unmodified chymotryptic IAPP under the conditions tested.

### 3.2. Cu(II)-Mediated Oxidation Impacts the Kinetics of Amyloid Self-Assembly

**Figure 2:**
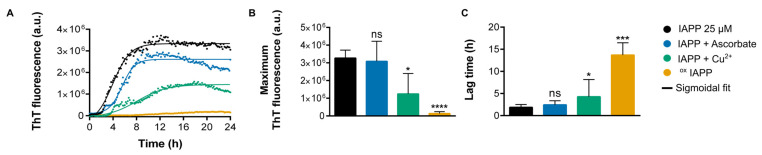
Assessment of IAPP amyloid self-assembly in the presence or absence of Cu(II) and ascorbic acid. (**A**) Kinetics of IAPP amyloid formation at 25 µM monitored by ThT. (**B**) ThT fluorescence intensity at endpoint. (**C**) Lag time determined from sigmoidal fit. (**B**,**C**). Data were collected from at least four independent experiments performed in triplicates. *p* < 0.05 (*); *p* < 0.0005 (***); *p* < 0.0001 (****); ns: non-significant.

It has been previously demonstrated that oxidative modifications influence the self-assembly behavior of amyloidogenic polypeptides, such as Aβ [49,50] and α-synuclein [51]. Thus, the impact of copper-mediated oxidation on IAPP aggregation into amyloid fibrils was assessed. Amyloid fibril formation was monitored using the fluorescence of ThT, a dye that exhibits enhanced signal upon interaction with cross-β-sheet quaternary structures [52]. Figure 2 displays the aggregation kinetics of IAPP in absence or in presence of, respectively, copper, ascorbic acid, and both reagents that induce IAPP oxidation.

The addition of 10 molar eq. of ascorbic acid did not significantly affect the kinetics of IAPP self-assembly, as the ThT sigmoidal profile remained comparable to the control conditions. Under neutral pH conditions, IAPP efficiently forms amyloid fibrils in vitro, even at low micromolar concentrations [10]. In contrast, introducing 1 molar eq. of copper led to a clear delay in fibrillization, indicating that copper interacts with IAPP proteospecies and modulates the aggregation pathway (Figure 2A). This delay in fibril formation in the presence of copper is consistent with previous findings reported [23] and supports the modulatory effect of copper on IAPP aggregation. Most notably, the presence of both copper and ascorbic acid, which readily induces the oxidation of IAPP as confirmed by mass spectrometry (Figure 1), significantly prolonged the lag phase before fibril formation, extending it to approximately 12 h, three times longer than the 3-h lag phase observed for the non-oxidized counterpart (Figure 2C and Table S2). Moreover, the final ThT fluorescence was decreased significantly under oxidative conditions, suggesting a lower content of structures with a well-defined cross-β-sheet architecture (Figure 2C and Table S2).

### 3.3. Copper-Induced Oxidation Promotes the Formation of Amorphous Aggregates

**Figure 3:**
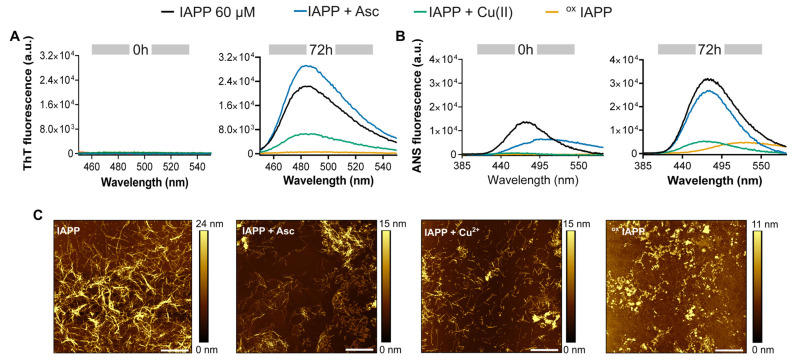
Analysis of the effect of oxidation on IAPP assemblies. (**A**–**C**) IAPP was incubated at 60 µM at room temperature for 72 h in the presence of Cu(II) (50 µM) and/or ascorbic acid (500 µM). The mixture was analyzed by: (**A**) ThT fluorescence with excitation at 440 nm, (**B**) ANS fluorescence with excitation at 355 nm, and (**C**) AFM: Scale bar is 2 µm and each image is 10 μm × 10 μm.

To gain further insights on the effects of oxidation on amyloid formation observed with ThT kinetics, the resulting supramolecular organization was analyzed (Figure 3). To do so, IAPP was incubated under fully quiescent conditions (pH 7.4) in phosphate buffer for 72 h in presence, or not, of copper and/or ascorbic acid. Considering that the recurrent displacements of the microplate within the fluorimeter induce significant agitation (Figure 2), which promotes amyloid formation by mass transport and fibril fragmentation [40], self-assembly under fully quiescent conditions was evaluated at an IAPP concentration of 60 μM, compared to 25 μM for the ThT kinetics assay. ThT fluorescence spectra recorded for oxidized IAPP showed markedly reduced signal, suggesting a structural organization that is less compatible with strong dye binding compared to the non-oxidized species (Figure 3A). In parallel, ANS fluorescence spectra recorded for oxidized IAPP exhibit a reduced signal accompanied by a red shift, with a fluorescence maximum at 528 nm compared to approximately 470 nm for the non-oxidized peptide (Figure 3B). These results suggest that oxidized IAPP displays lower surface-accessible hydrophobicity and follows a different aggregation mechanism than the non-oxidized species [53].

Figure 3C presents the morphological characterization of the aggregates by atomic force microscopy (AFM). IAPP incubated in the presence of ascorbic acid assembled into long, well-defined fibrils that resemble the twisted and flat ribbon-like structures characteristic of amyloid quaternary assemblies [54]. Conversely, samples incubated with copper alone exhibited shorter fibrils, whereas oxidized IAPP predominantly formed amorphous, non-fibrillar aggregates. Similar morphologies were observed for IAPP incubated for shorter period, i.e., 24 h (Figure S2). These morphological differences align with the altered dye-binding properties observed by fluorescence spectroscopy. Unfortunately, circular dichroism spectroscopy, which is commonly employed to probe the secondary conformational shift in IAPP associated with its aggregation [55], could not be used because of the interference of the oxidative mixture with the CD signal at shorter wavelength.

### 3.4. Oxidized IAPP Aggregates Do Not Seed Amyloid Formation

**Figure 4:**
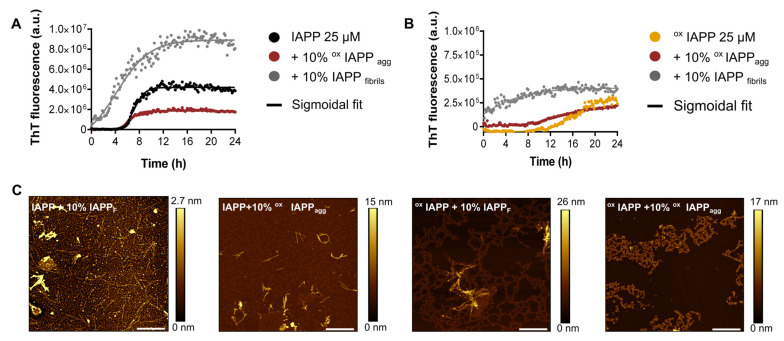
Evaluation of the seeding capacity of oxidized IAPP aggregates. (**A**) Aggregation kinetics of 25 µM IAPP in the presence, or absence, of 10% preformed IAPP fibrils or oxidized IAPP aggregates. (**B**) Aggregation kinetics of 25 µM oxidized IAPP in the presence or absence of 10% preformed IAPP fibrils or oxidized IAPP aggregates. (**A**,**B**) Peptides were incubated at 25 µM in 50 mM phosphate buffer, pH 7.4, with or without seeds, and ThT fluorescence was measured using an excitation wavelength of 440 nm. Curves were fitted using a sigmoidal growth model. (**C**) AFM imaging after 72 h of incubation. Scale bar is 2 µm and each image is 10 μm × 10 μm.

Considering the divergence in the supramolecular organization of the aggregated structures obtained for oxidized IAPP, we evaluated their capacity to induce the aggregation of unmodified IAPP with a seeding experiment. Seeding, similar to a prion-like effect, has long attracted interest, as it represents a hallmark of pathological processes involved in protein misfolding disorders [56]. Amyloid seeds were prepared by incubating either oxidized, or non-oxidized IAPP, for 72 h at room temperature without agitation. Next, the aggregation kinetics of 25 µM IAPP, or oxidized IAPP, in presence or in absence of these preformed assemblies, were monitored by ThT fluorescence over 24 h. As anticipated, the addition of 10% preformed unmodified IAPP fibrils markedly accelerated IAPP amyloid self-assembly, with the quasi-disappearance of the lag-phase (Figure 4A,B). In contrast, seeding unmodified IAPP with pre-aggregated oxidized IAPP had no significant effect on the aggregation kinetics of IAPP, either under oxidation conditions or not (Figure 4A,B).

The seeding effect of pre-assembled fibrils was further investigated by analyzing the final fibril morphology using AFM (Figure 4C). The AFM images of unmodified IAPP seeded with preformed IAPP fibrils revealed predominantly long, twisted, and homogeneous fibrils (Figure 3C). In contrast, seeding unmodified IAPP with preformed oxidized IAPP aggregates resulted in shorter fibrils with heterogeneous sizes. Moreover, the assembly of oxidized IAPP, i.e., incubated with Cu and ascorbic acid, in the presence of 10% IAPP fibrils or oxidized IAPP aggregates mainly led to the formation of amorphous aggregates. These observations suggest that under oxidative conditions, IAPP self-assembles into mainly amorphous aggregates, even in presence of well-defined amyloid seeds. A plausible hypothesis is that oxidative modifications induced by copper alter specific molecular interactions within IAPP, thereby delaying or disrupting the critical steps required for the ordered formation of amyloid self-assembly.

### 3.5. Substitution of IAPP His18 by Ala Alters the Oxidation Pattern

**Figure 5:**
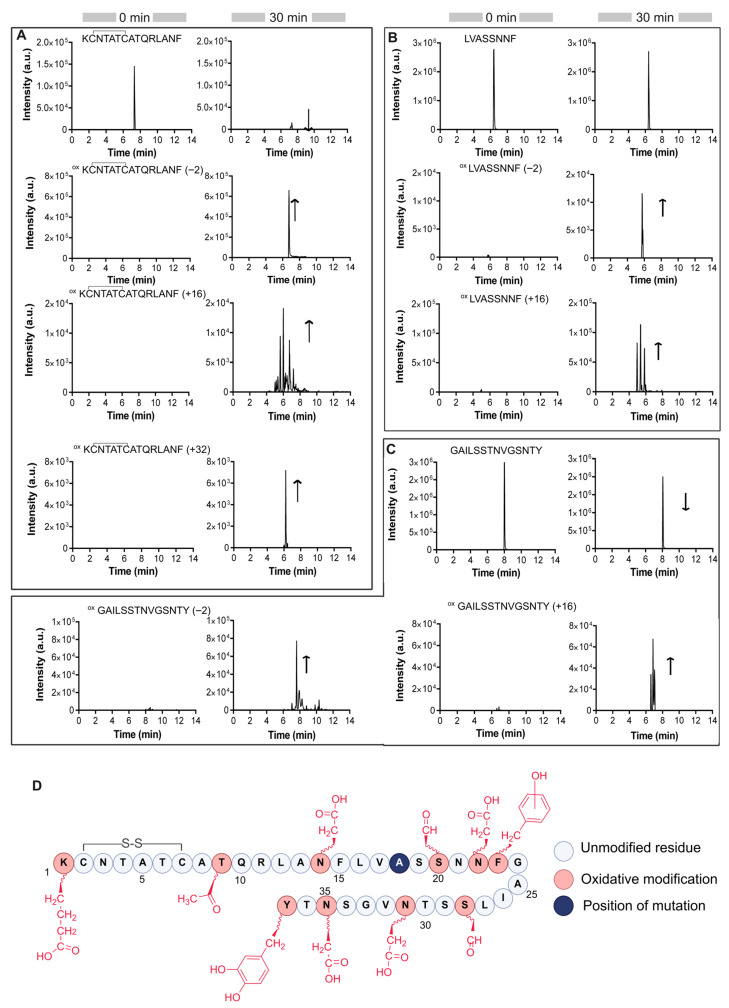
Trace chromatograms of chymotryptic IAPP H18A before and after 30 min oxidation using Cu/O_2_/Asc/ system at room temperature. (**A**) **^1^**KCNTATCATQRLANF**^15^**, (**B**) **^16^**LVHSSNNF**^23^** and (**C**) **^24^**GAILSSTNVGSNTY**^37^**. The arrows specify the trend of chromatographic peaks. Mass accuracy and retention time assigned to each peak are presented in Table S3. (**D**) Summary of Cu-catalyzed oxidative modifications on [H18A]IAPP. The His-to-Ala substitution is highlighted in dark blue, and oxidative modification sites identified by MS/MS analysis are shown in red.

Previous studies have demonstrated that the histidine imidazole sidechain plays a critical role for copper coordination and subsequent oxidation [57,58]. Thus, the histidine 18 of IAPP was substituted by an alanine to assess the impact of a change in copper coordination (loosely bound copper) on IAPP oxidation using the same UPLC-MS/MS workflow applied to the wild-type (WT) peptide. Figure 5 presents the extracted ion chromatograms of chymotryptic peptides from the H18A derivative before and after 30 min copper-mediated oxidation. Representative MS/MS spectra are provided in the Supplementary Information (Figure S3). Compared to WT IAPP, chromatograms revealed an increased diversity of oxidized peptide signals and complexity for IAPP H18A, in agreement with a metal-catalyzed oxidation driven by a loosely bound copper. It is worth mentioning that the H18A substitution, in contrast to the S20G mutation that is associated with early-onset diabetes by increasing amyloidogenicity [59,60], is not a naturally occurring mutation. It is simply used in this study as a chemical tool to probe the contribution of His sidechain on copper-mediated oxidation.

Analysis of the first chymotryptic peptide from [H18A]IAPP_(1–15)_ revealed a noticeable increase in the number of the oxidation products compared to its WT counterpart. Specifically, the extracted ion chromatogram corresponding to a +16 Da mass shift, indicative of the addition of a single oxygen atom, displayed several distinct peaks with identical *m/z* values but different retention times (Table S3). This pattern indicates the presence of positional isomers arising from oxidation at multiple sites. A similar multiplicity of oxidized species was also observed for the [H18A]IAPP_(16–23)_ and [H18A]IAPP_(24–37)_ peptides. Figure 5 provides an overview of the oxidized residues identified by MS/MS analysis (see also Figure S3). In addition to oxidative modifications previously observed in certain residues of WT IAPP, MS/MS analysis of oxidized [H18A]IAPP revealed novel oxidative reactions, including the oxidation of lysine to aminoadipic acid, phenylalanine to tyrosine, and tyrosine to L-DOPA [61,62,63].

### 3.6. Oxidation of [H18A]IAPP Modulates Amyloid Formation

Next, the impact of the widespread oxidation of IAPP H18A on its ability to self-assemble was assessed using ThT fluorescence and AFM imaging. In comparison to WT IAPP, the H18A derivative displayed accelerated aggregation kinetics, with no detectable lag phase. Notably, the presence of copper did not alter the rate of aggregation, which remained comparable to that observed in its absence. This suggests that the absence of histidine at position 18 eliminates the primary copper-binding site, thereby changing the copper coordination sphere and its associated modulatory effects on IAPP aggregation [64]. Consequently, the aggregation kinetics of [H18A]IAPP remained unaffected by copper, in contrast to WT IAPP where Cu(II)–His18 interactions significantly alter the aggregation pathway.

**Figure 6:**
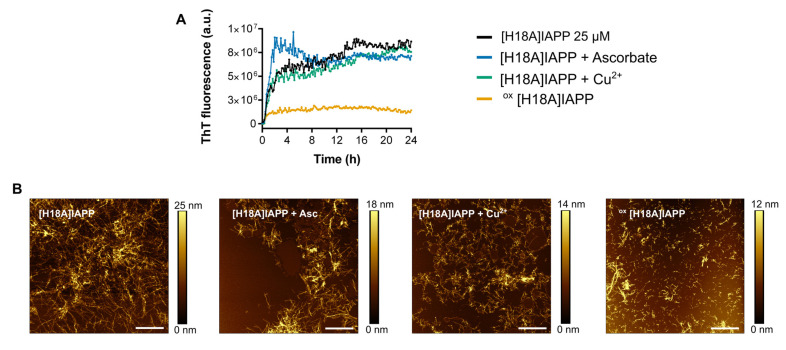
(**A**) Self-assembly of [H18A]IAPP (25 µM) monitored by ThT fluorescence in the presence of copper (25 µM), and/or ascorbate (250 µM), in phosphate buffer (pH 7.4). (**B**) AFM images of [H18A]IAPP (60 µM) in the presence, or absence, of copper and ascorbate, after pre-incubation under quiescent conditions for 72 h prior to analysis. Scale bar is 2 µm and each image is 10 μm × 10 μm.

Furthermore, oxidized [H18A]IAPP rapidly formed ThT-positive fibrils, as evidenced by AFM imaging. After 72 h of fully quiescent incubation, short fibrils were clearly visible for oxidized [H18A]IAPP (Figure 6), whereas WT IAPP predominantly formed amorphous aggregates under similar conditions (Figure 3). These observations suggest that substituting histidine with alanine at position 18 promotes fibril formation and bypasses copper-induced modulation of aggregation. The absence of this key metal-binding residue appears to promote organized fibrillization, despite the heterogeneous nature of oxidative modifications.

## 4. Conclusions

This study revealed that copper-catalyzed oxidation provides an alternative pathway that precludes the formation of well-ordered IAPP fibrils by promoting the accumulation of amorphous aggregates. These results shed light on the interrelation between oxidative modifications and amyloid self-assembly, highlighting how redox-active metals such as copper can modulate the aggregation outcome of amyloidogenic peptides. The identification of oxidized residues within the amyloidogenic core region further emphasizes the critical role of site-specific modifications in altering aggregation behavior. Moreover, the H18A substitution, which led to loosely bound copper, promoted distinct oxidation patterns and enabled fibril formation even under oxidative conditions. Together, these findings revealed that the presence or absence of a key copper-binding residue, such as histidine, can dramatically alter the conformational ensemble of IAPP under oxidative stress. An important open question, however, is whether the amorphous aggregates produced by copper-mediated oxidation are intrinsically toxic to pancreatic β-cells and thus contribute to the progression of type 2 diabetes, or whether this oxidative pathway instead represents a cellular defense mechanism designed to limit amyloid-driven toxicity.

## Figures and Tables

**Table 1:** Theoretical and experimental monoisotopic *m/z* values, mass accuracies, and retention times are presented for chymotryptic IAPP peptides in both their non-oxidized and oxidized forms. Detected oxidative modifications are indicated in parentheses, where −2, +16, and +32, respectively, correspond to the loss of two hydrogen atoms, the addition of one oxygen atom, and the addition of two oxygen atoms.

Peptide	Charge	*m*/*z* (Exact)	*m*/*z* (Measured)	Error (ppm)	Retention Time (min)
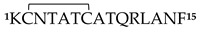	+3	547.2613	547.2610	−0.56	7.27
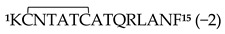	+3	546.5895	546.5891	0.44	6.71
^**16**^LVHSSNNF^**23**^	+2	459.2279	459.2282	0.85	4.29
^**16**^LVHSSNNF^**23**^ (−2)	+2	458.2201	458.2197	0.44	4.69
^**16**^LVHSSNNF^**23**^ (+16)	+2	467.2253	467.2244	−1.0	5.90
^**16**^LVHSSNNF^**23**^ (+32)	+2	475.2228	475.2226	0.70	5.70
^**24**^GAILSSTNVGSNTY^**37**^	+2	692.3416	692.3420	0.09	7.98
^**24**^GAILSSTNVGSNTY^**37**^ (+16)	+2	700.3391	700.3393	1.0	6.81

## Data Availability

The original contributions presented in this study are included in the article or Supplementary Materials. Further inquiries can be directed to the corresponding authors.

## Supplementary Materials

The following supporting information can be downloaded at: https://www.mdpi.com/article/10.3390/antiox14111269/s1; Table S1: Mass spectrometry data of miss-cleaved IAPP peptides; Figure S1: Annotated MS/MS spectra of native and oxidized IAPP; Table S2: Parameters from sigmoidal fits using Boltzmann equation for fitted curves of IAPP; Figure S2: AFM images obtained after pre-incubation under quiescent conditions for 24 h; Table S3: Mass spectrometry data of [H18A]IAPP peptides; Figure S3: Annotated MS/MS spectra of native and oxidized [H18A]IAPP.
