# Supplementary material for: Effect of Copper-Catalyzed Oxidation on the Aggregation of the Islet Amyloid Polypeptide
Source: Antioxidants (Basel). 2025 Oct 22;14(11):1269. doi: 10.3390/antiox14111269 (PMC12649479; doi:10.3390/antiox14111269)
Supplement: Supplementary file 1 [file antioxidants-14-01269-s001.zip › antioxidants-3906739-supplementary.pdf]

SUPPORTING INFORMATION

Effect of Copper-Catalyzed Oxidation on the Aggregation of the Islet Amyloid Polypeptide

Océane Amilca [1,2,3], Phuong Trang Nguyen [1,2], Lucie Perquis [3], Fabrice Collin [3,*] and Steve Bourgault [1,2,*]

[1] Department of Chemistry, Université du Québec à Montréal, Montreal, QC H3C 3P8, Canada
[2] Quebec Network for Research on Protein Function, Engineering and Applications (PROTEO), Montreal, QC H3C 3P8, Canada
[3] Laboratoire Softmat, Université de Toulouse, CNRS UMR 5623, 31400 Toulouse, France
* Correspondence: fabrice.collin@utoulouse.fr (F.C.); bourgault.steve@uqam.ca (S.B.)

Table S1. Theoretical and experimental monoisotopic m/z values, mass accuracies, and retention times for unmodified and oxidized IAPP peptides digested with α-chymotrypsin. Detected oxidative modifications are indicated in parentheses: +16, +32, +48 and +0.98 correspond to the addition of one oxygen atom, two oxygen atoms, three oxygen atoms and deamidation, respectively.

Peptide	Charge	m/z (exact)	m/z (measured)	Error (ppm)	Retention time (min)
^{13}ANFLVHSSNNF^{23}	+2	625.3013	625.3018	0.8	8.04
^{13}ANFLVHSSNNF^{23} (+16)	+2	633.2996	633.2998	0.2	7.28
^{13}ANFLVHSSNNF^{23} (+32)	+2	641.2974	641.2953	-3.3	10.12
^{15}FLVHSSNNF^{23}	+2	532.7610	532.7620	1.8	7.20
^{15}FLVHSSNNF^{23} (+16)	+2	540.7598	540.7597	-0.1	8.88
^{16}LVHSSNNFGAIL^{27}	+3	424.5633	424.5643	2.2	9.04
^{16}LVHSSNNFGAIL^{27} (+16) / (+0.98)	+2	644.8296	644.8316	3.1	9.28
^{16}LVHSSNNFGAIL^{27} (+16)	+2	644.3383	644.3388	0.8	8.98
^{16}LVHSSNNFGAIL^{27} (+32)	+2	652.3370	652.3352	-2.8	10.81
^{16}LVHSSNNFGAIL^{27} (+48)	+2	660.3350	660.3342	-1.3	6.96
^{17}VHSSNNF^{23}	+2	402.6874	402.6868	-1.6	3.13
^{17}VHSSNNF^{23} (+16)	+2	410.6830	410.6835	1.1	4.15
^{17}VHSSNNFGAIL^{27}	+2	579.8001	579.7995	-1.1	7.20
^{17}VHSSNNFGAIL^{27} (+16)	+2	587.7965	587.7955	-1.7	8.17
^{17}VHSSNNFGAIL^{27} (+32)	+2	595.7938	595.7938	-0.1	9.94
^{17}VHSSNNFGAIL^{27} (+48)	+2	603.7909	603.7902	-1.1	6.18
^{24}GAILSSTNVGSNTY^{37} (0.98)	+2	691.8449	691.8470	-3.0	7.57
^{24}GAILSSTNVGSNTY^{37} (+32) / (+0.98)	+2	699.8465	707.8419	-2.0	7.89

A

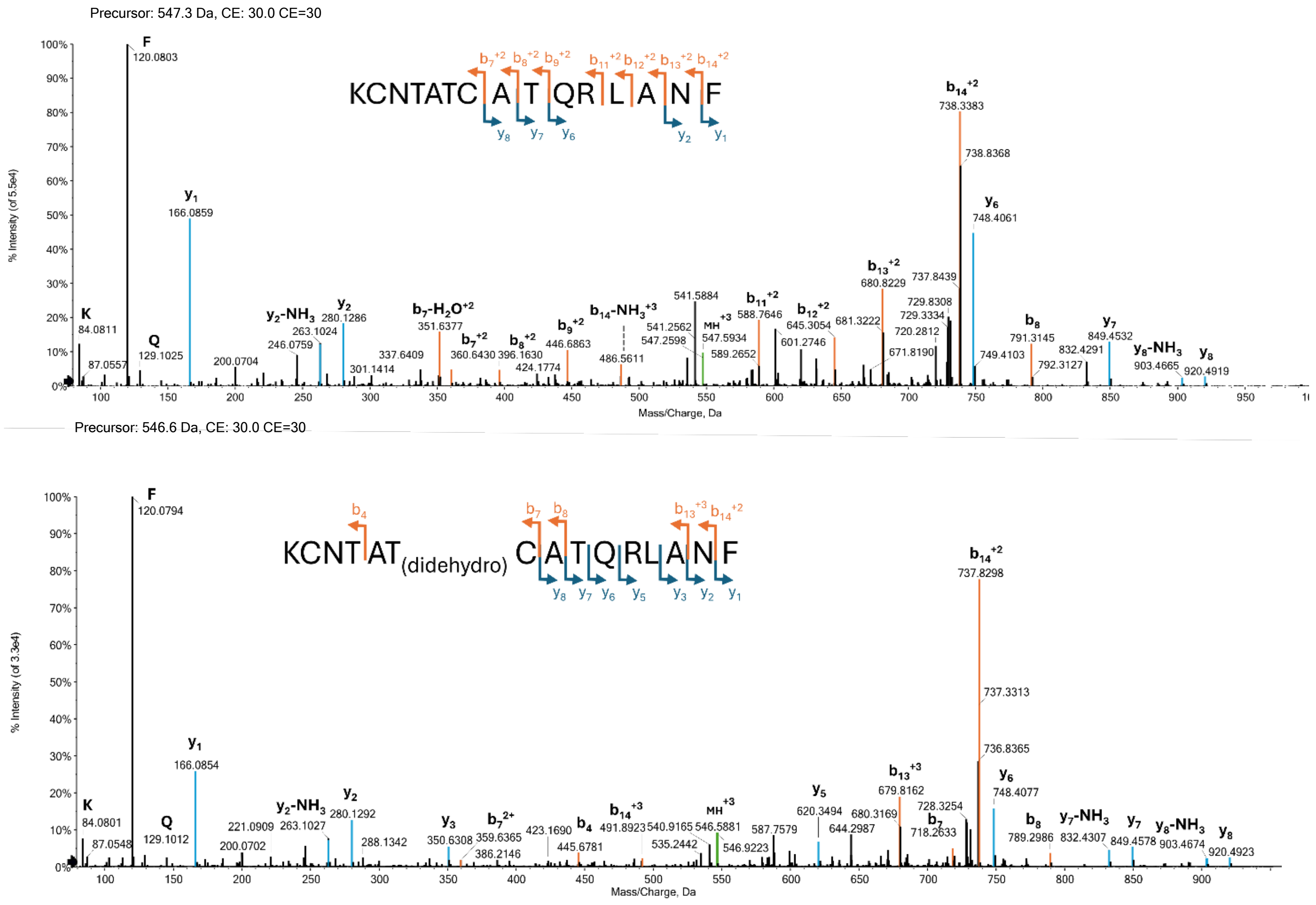

Precursor: 547.3 Da, CE: 30.0 CE=30
KCNTATCATQRLANF
% Intensity (of 5.5e4)
Mass/Charge, Da
Precursor: 546.6 Da, CE: 30.0 CE=30
KCNTAT(didehydro)CATQRLANF
% Intensity (of 3.3e4)
Mass/Charge, Da

B

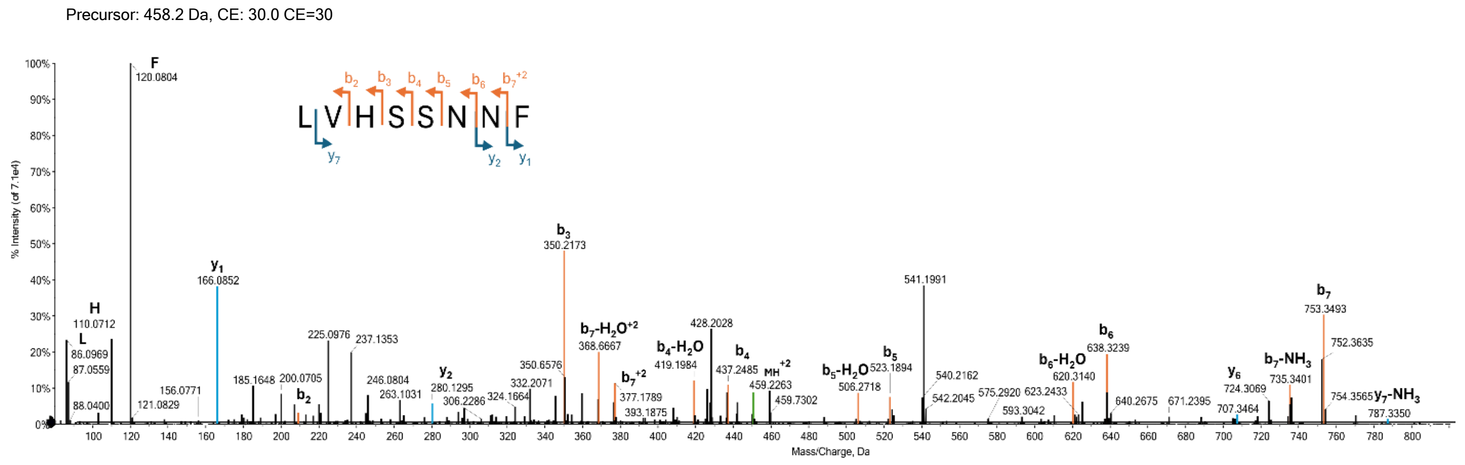

Precursor: 458.2 Da, CE: 30.0 CE=30
LVHSSNNF
% Intensity (of 7.1e4)
Mass/Charge, Da

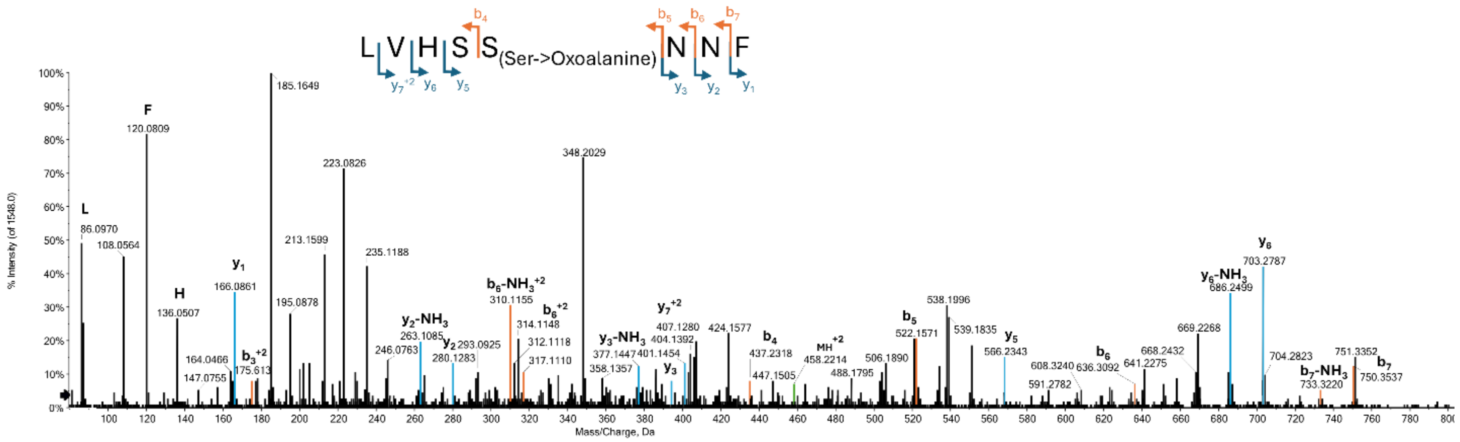

Precursor: 467.2 Da, CE: 30.0 CE=30

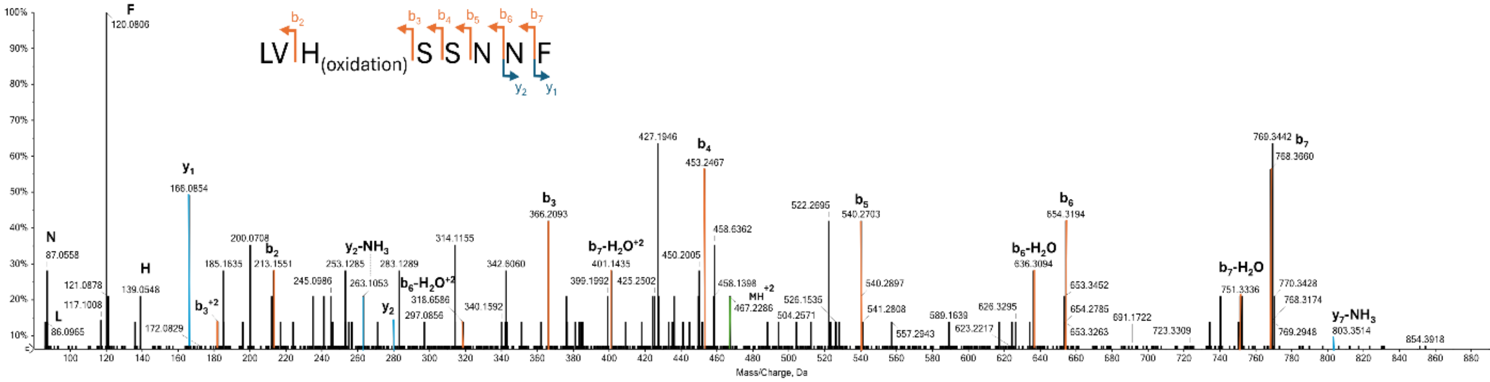

Precursor: 475.2 Da, CE: 30.0 CE=30

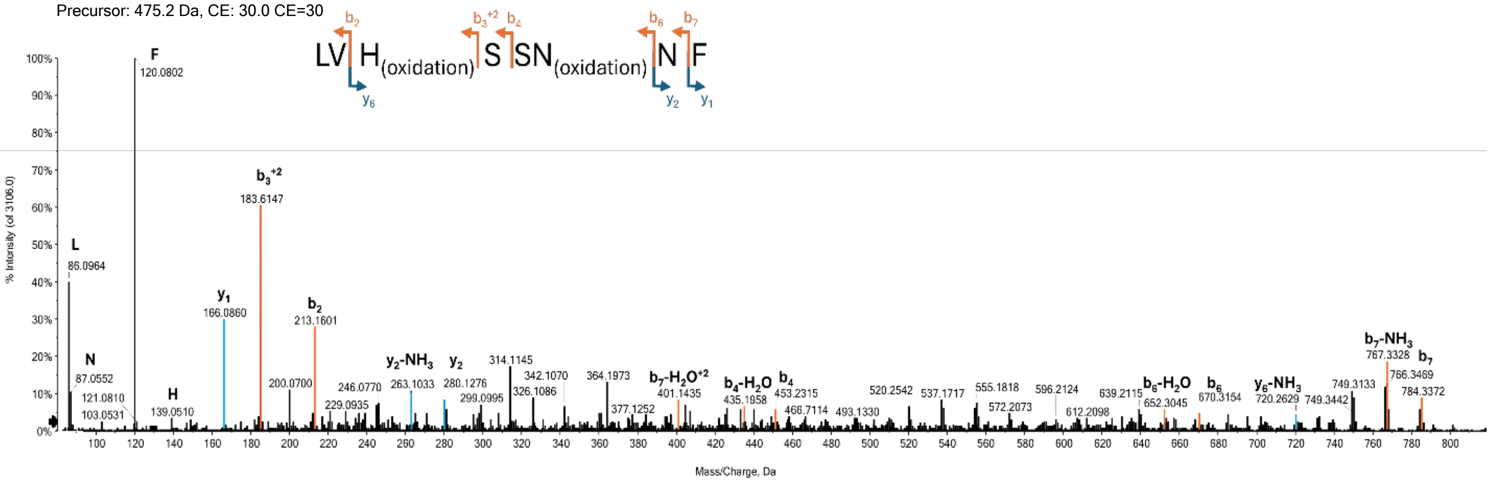

C

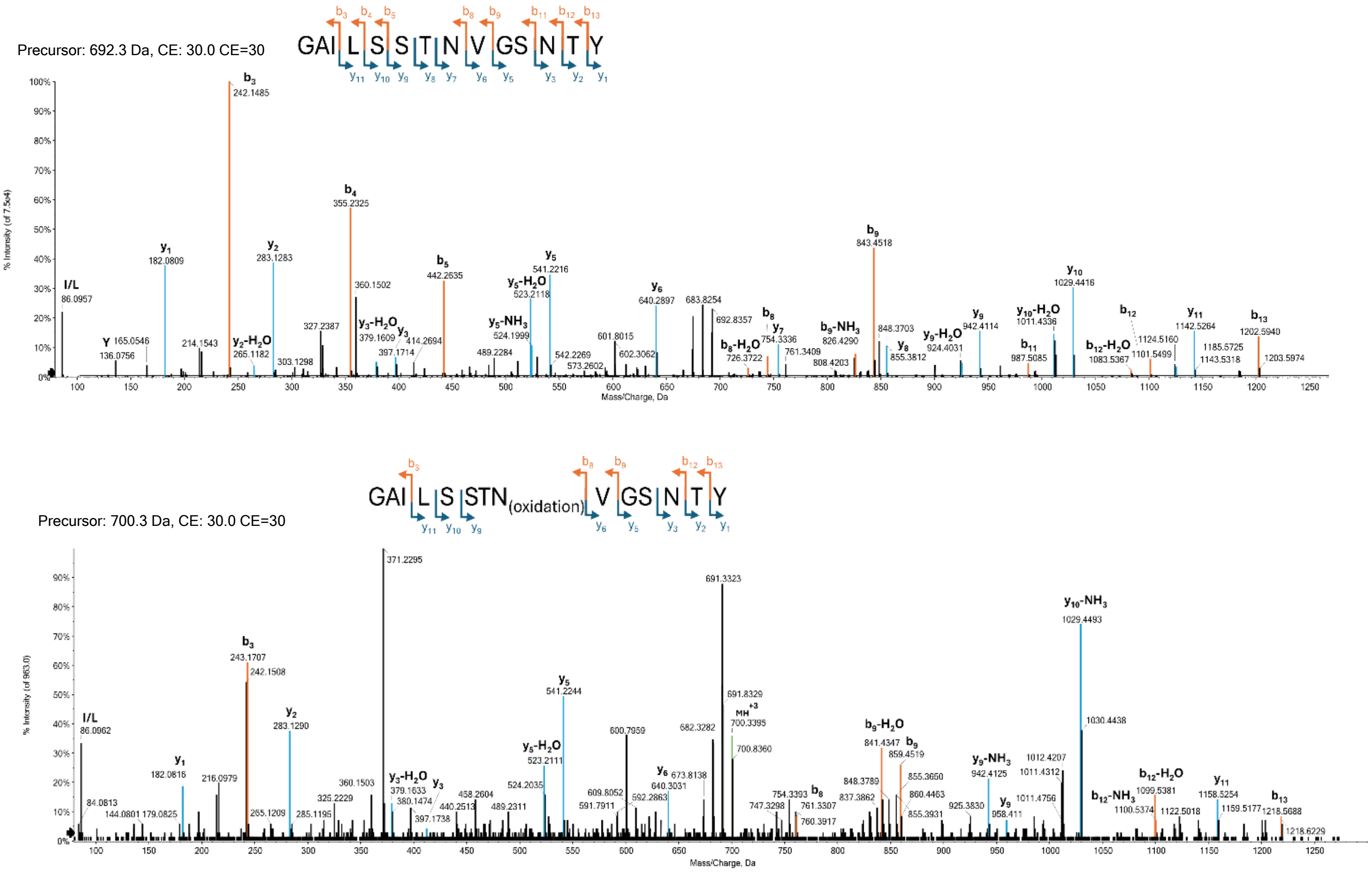

Figure S1. Identification of chymotryptic IAPP oxidation sites by LC-MS/MS. Annotated high-resolution MS/MS spectra of (**A**) ^{1}KCNTATCATQRLANF^{15}, (**B**) ^{16}LVHSSNNF^{23} and (**C**) ^{24}GAILSSTNVGSNTY^{37} peptides in their native and oxidized forms. Series of b- and y- ions are labeled in orange and blue, respectively. Precursor ions are highlighted in green and indicated above the spectra when detected. Spectra were acquired in CID mode (collision energy: 30 ± 10 V; cycle time: 1.05 s) with precursor charge states of z =+3 for IAPP$_{(1-15)}$ and z =+2 for IAPP$_{(16-23)}$ and IAPP$_{(24-37)}$. Fragment assignments were made with a mass tolerance of ± 5 ppm.

Table S2. Parameters obtained from sigmoidal fits using Boltzmann equation for fitted curves of IAPP as the means of at least four independent experiments performed in triplicates.

	Fluorescence (Y_{max})	Half time ($t_{1/2}$)	Lag time (t_{lag})
IAPP	3.27×10^6	3.561 ± 0.169	3.132
IAPP + Ascorbate	3.08×10^6	5.525 ± 0.20	3.433
IAPP + Cu^{2+}	1.24×10^6	8.104 ± 0.108	4.273
^{ox}IAPP	1.34×10^5	13.18 ± 0.2435	13.691

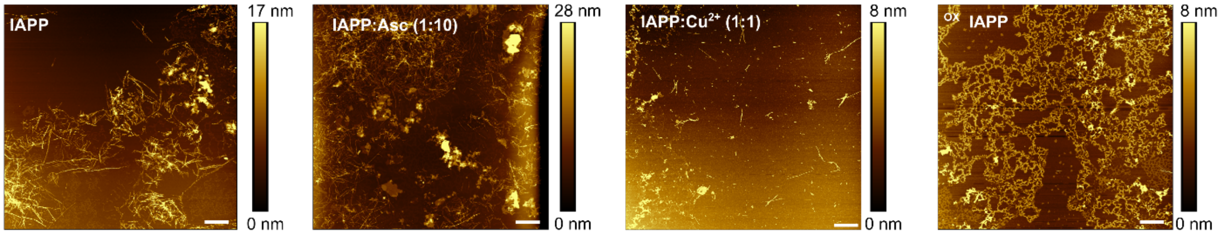

Figure S2. AFM images of IAPP (60 μM) in the presence, or absence, of copper and ascorbate, after pre-incubation under quiescent conditions for 24 h prior to analysis. Scale bar is 1 μm and scan frame is 10 μm × 10 μm.

Table S3. Theoretical and experimental monoisotopic m/z values, mass accuracies, and retention times for native [H18A]IAPP and oxidized [H18A]IAPP peptides digested with α-chymotrypsin. Detected oxidative modifications are indicated in parentheses, where –2, +16, and +32 respectively correspond to the loss of two hydrogen atoms, the addition of one oxygen atom, and the addition of two oxygen atoms.

Peptide	Charge	m/z (exact)	m/z (measured)	Error (ppm)	Retention time (min)
^{1}KCNTATCATQRLANF^{15}	+3	547.2613	547.2636	4.2	7.27
^{1}KCNTATCATQRLANF^{15} (-2)	+3	546.5895	546.5895	0	6.72
^{1}KCNTATCATQRLANF^{15} (+16)	+3	552.5930	552.5938	1.4	5.63/ 6.0/ 6.72/ 7.19
^{1}KCNTATCATQRLANF^{15} (+32)	+3	557.9246	557.9235	-1.97	6.17
^{16}LVASSNNF^{23}	+2	426.2170	426.283	3.05	6.39
^{16}LVASSNNF^{23} (-2)	+2	425.2092	425.2103	2.58	4.95/ 5.35/ 5.81
^{16}LVASSNNF^{23} (+16)	+2	434.2144	434.2138	-1.38	5.73
^{24}GAILSSTNVGSNTY^{37}	+2	692.3416	692.3427	1.58	7.98
^{24}GAILSSTNVGSNTY^{37} (-2)	+2	691.8378	691.8372	-0.86	6.59/ 6.93/ 7.03
^{24}GAILSSTNVGSNTY^{37} (+16)	+2	700.3391	700.3386	-0.71	7.58

A

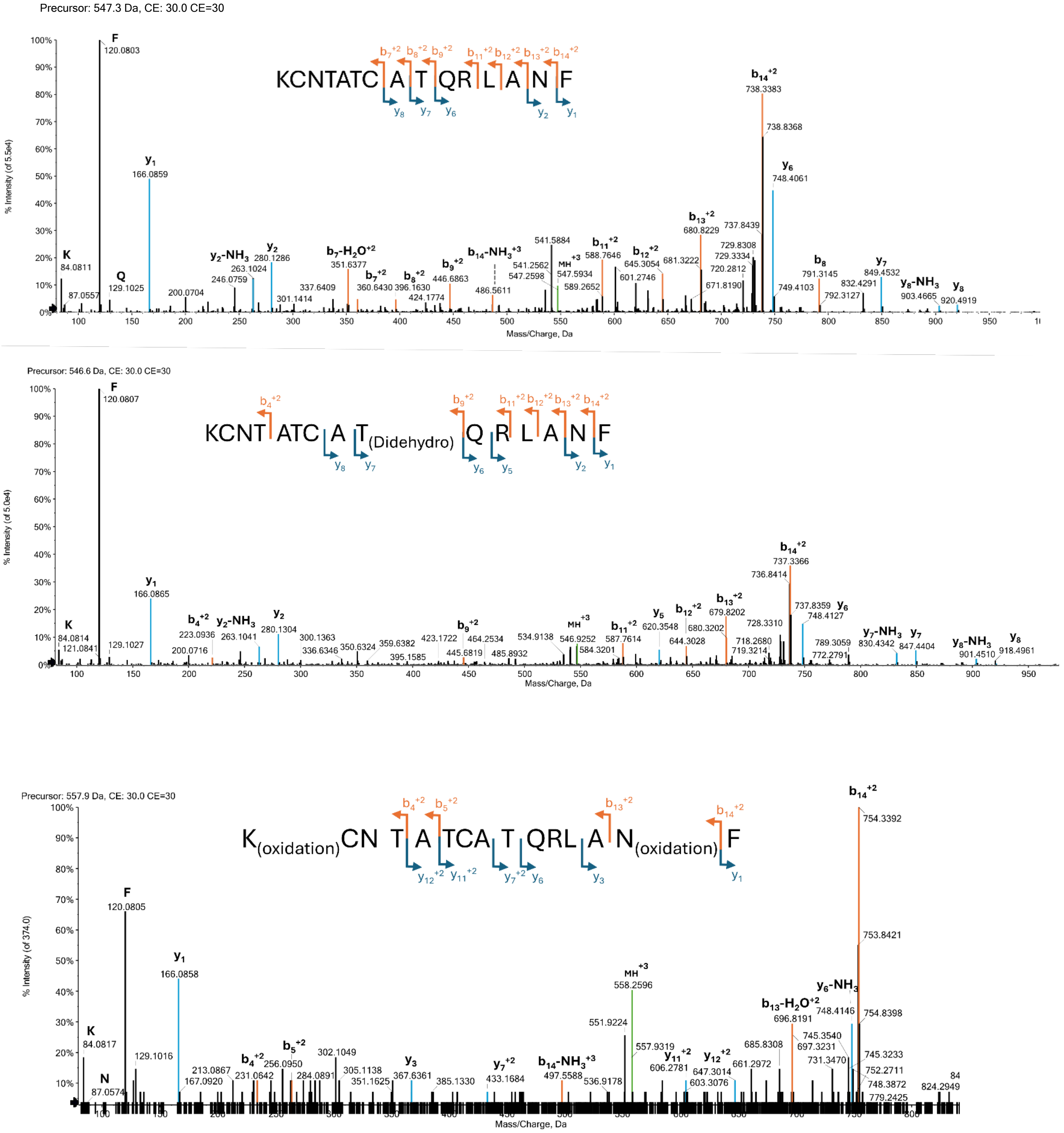

Precursor: 547.3 Da, CE: 30.0 CE=30
KCNTATCATQRLANF
% Intensity (of 5.5e4)
Mass/Charge, Da
Precursor: 546.6 Da, CE: 30.0 CE=30
KCNTATCAT(Didehydro)QRLANF
% Intensity (of 5.0e4)
Mass/Charge, Da
Precursor: 557.9 Da, CE: 30.0 CE=30
K(oxidation)CNTATCATQRLAN(oxidation)F
% Intensity (of 374.0)
Mass/Charge, Da

B

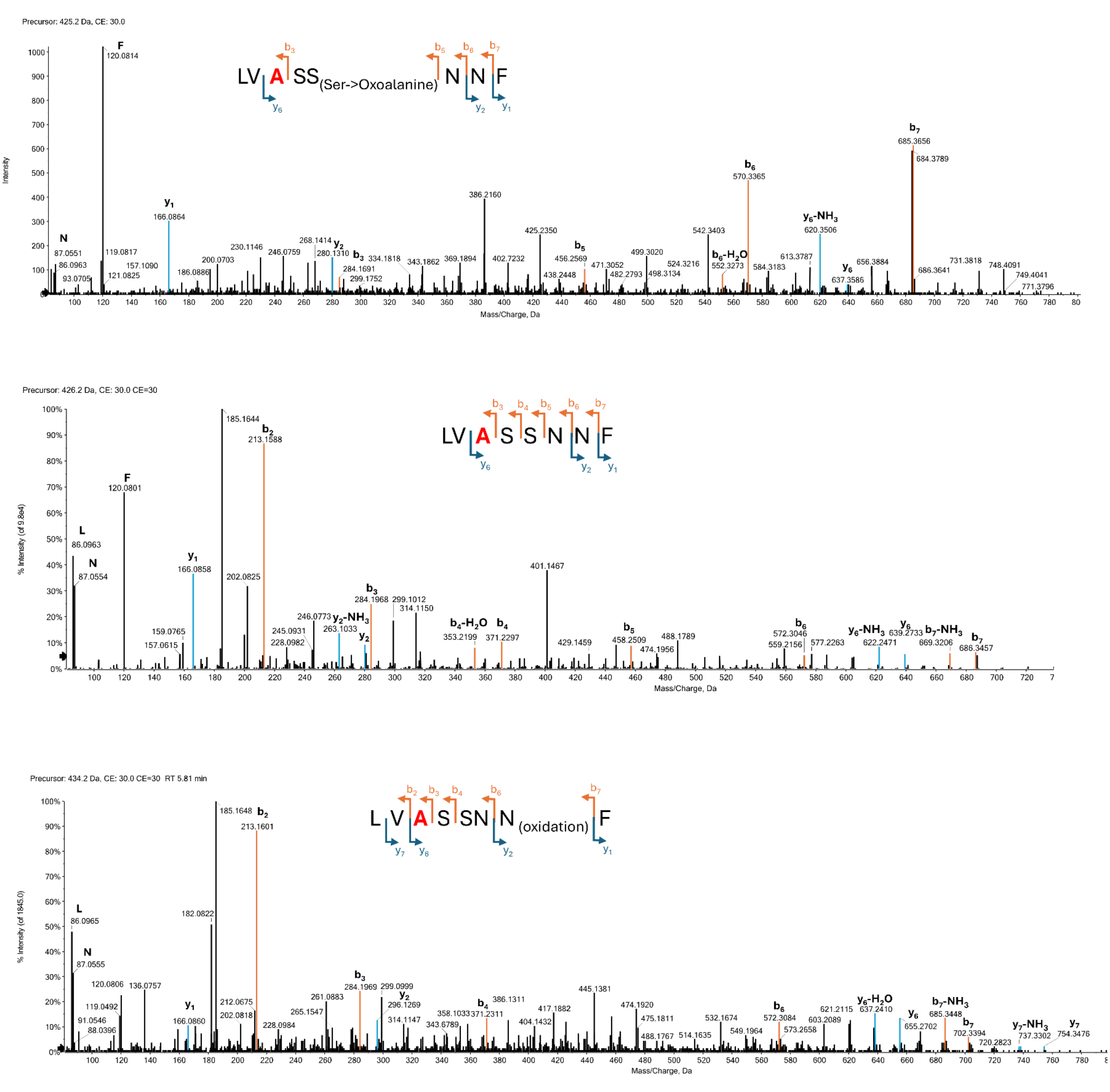

Precursor: 425.2 Da, CE: 30.0
LV A SS(Ser->Oxoalanine) N N F
b3 b5 b6 b7
y6 y2 y1
Intensity
Mass/Charge, Da
F 120.0814
N 87.0551
86.0963
93.0705
119.0817
121.0825
157.1090
y1 166.0864
186.0886
200.0703
230.1146
246.0759
268.1414
y2 280.1310
b3 284.1691
299.1752
334.1818
343.1862
369.1894
386.2160
402.7232
425.2350
438.2448
b5 456.2569
471.3052
482.2793
498.3134
499.3020
524.3216
542.3403
b6-H2O 552.3273
b6 570.3365
584.3183
613.3787
y6-NH3 620.3506
y6 637.3586
656.3884
b7 685.3656
684.3789
686.3641
731.3818
748.4091
749.4041
771.3796
Precursor: 426.2 Da, CE: 30.0 CE=30
LV A S S N N F
b3 b4 b5 b6 b7
y6 y2 y1
% Intensity (of 9.8e4)
Mass/Charge, Da
L 86.0963
N 87.0554
F 120.0801
157.0615
159.0765
y1 166.0858
185.1644
202.0825
b2 213.1588
228.0982
245.0931
246.0773
y2-NH3 263.1033
y2
b3 284.1968
299.1012
314.1150
b4-H2O 353.2199
b4 371.2297
401.1467
429.1459
b5 458.2509
474.1956
488.1789
559.2156
b6 572.3046
577.2263
y6-NH3 622.2471
y6 639.2733
b7-NH3 669.3206
b7 686.3457
Precursor: 434.2 Da, CE: 30.0 CE=30 RT 5.81 min
L V A S SN N (oxidation) F
b2 b3 b4 b6 b7
y7 y6 y2 y1
% Intensity (of 1845.0)
Mass/Charge, Da
L 86.0965
N 87.0555
88.0396
91.0546
119.0492
120.0806
136.0757
y1 166.0860
182.0822
185.1648
b2 213.1601
202.0818
212.0675
228.0984
261.0883
265.1547
b3 284.1969
y2 296.1269
299.0999
314.1147
343.6789
358.1033
b4 371.2311
386.1311
404.1432
417.1882
445.1381
474.1920
475.1811
488.1767
514.1635
532.1674
549.1964
b6 572.3084
573.2658
603.2089
621.2115
y6-H2O 637.2410
y6 655.2702
b7-NH3 685.3448
b7 702.3394
720.2823
y7-NH3 737.3302
y7 754.3476

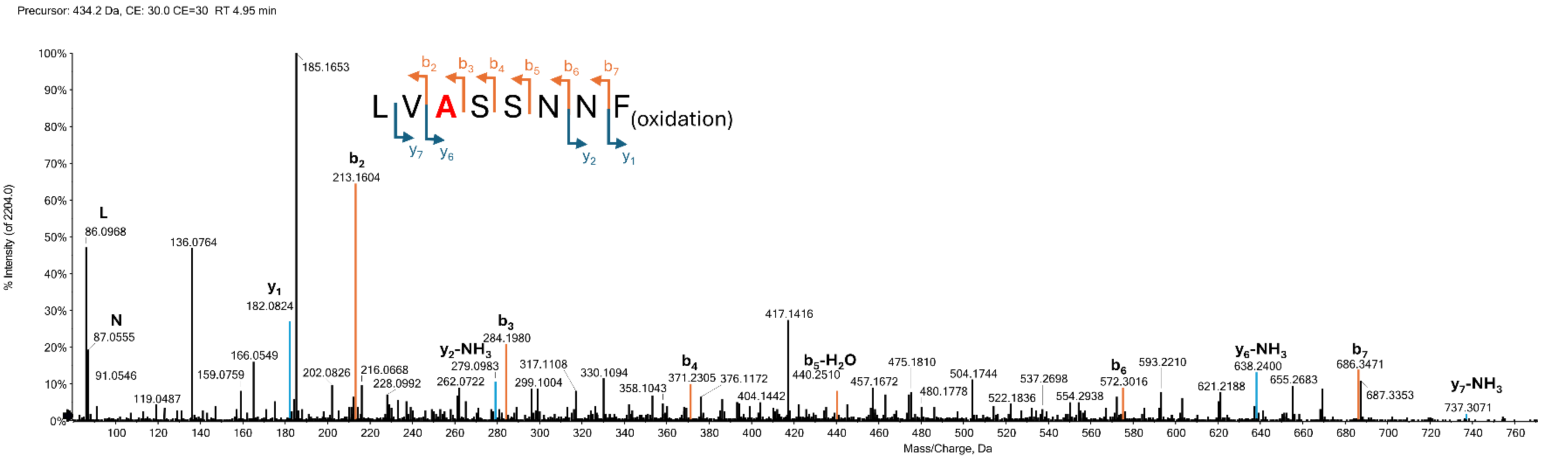

Precursor: 434.2 Da, CE: 30.0 CE=30 RT 4.95 min
L V A S S N N F(oxidation)
b2 b3 b4 b5 b6 b7
y7 y6 y2 y1
% Intensity (of 2204.0)
Mass/Charge, Da
L 86.0968
N 87.0555
91.0546
119.0487
136.0764
159.0759
166.0549
y1 182.0824
185.1653
202.0826
b2 213.1604
216.0668
228.0992
262.0722
y2-NH3 279.0983
b3 284.1980
299.1004
317.1108
330.1094
358.1043
b4 371.2305
376.1172
404.1442
417.1416
b5-H2O 440.2510
457.1672
475.1810
480.1778
504.1744
522.1836
537.2698
554.2938
b6 572.3016
593.2210
621.2188
y6-NH3 638.2400
655.2683
b7 686.3471
687.3353
y7-NH3 737.3071

C

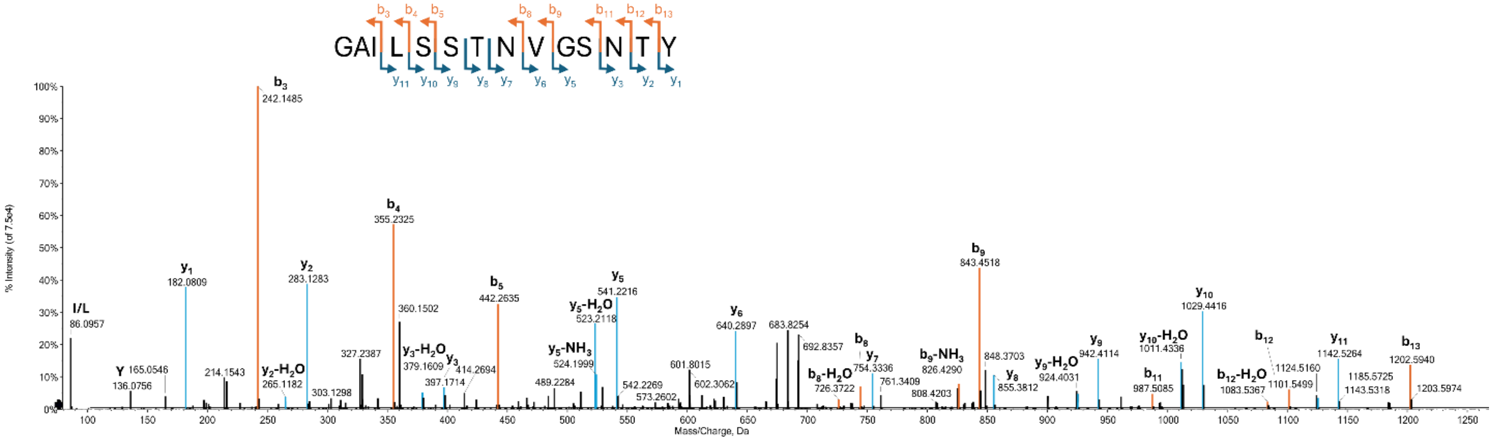

GAI L S S T N V GS N T Y
b3 b4 b5 b8 b9 b11 b12 b13
y11 y10 y9 y8 y7 y6 y5 y3 y2 y1
% Intensity (of 7.5e4)
Mass/Charge, Da
I/L 86.0957
Y 136.0756
165.0546
y1 182.0809
214.1543
b3 242.1485
y2-H2O 265.1182
y2 283.1283
303.1298
327.2387
b4 355.2325
360.1502
y3-H2O 379.1609
y3 397.1714
414.2694
b5 442.2635
489.2284
y5-NH3 524.1999
y5-H2O 523.2118
y5 541.2216
542.2269
573.2602
601.8015
602.3062
y6 640.2897
683.8254
692.8357
b8-H2O 726.3722
b8 y7 754.3336
761.3409
808.4203
b9-NH3 826.4290
b9 843.4518
848.3703
y8 855.3812
y9-H2O 924.4031
y9 942.4114
b11 987.5085
y10-H2O 1011.4336
y10 1029.4416
b12-H2O 1083.5367
b12 1101.5499
1124.5160
y11 1142.5264
1143.5318
1185.5725
b13 1202.5940
1203.5974

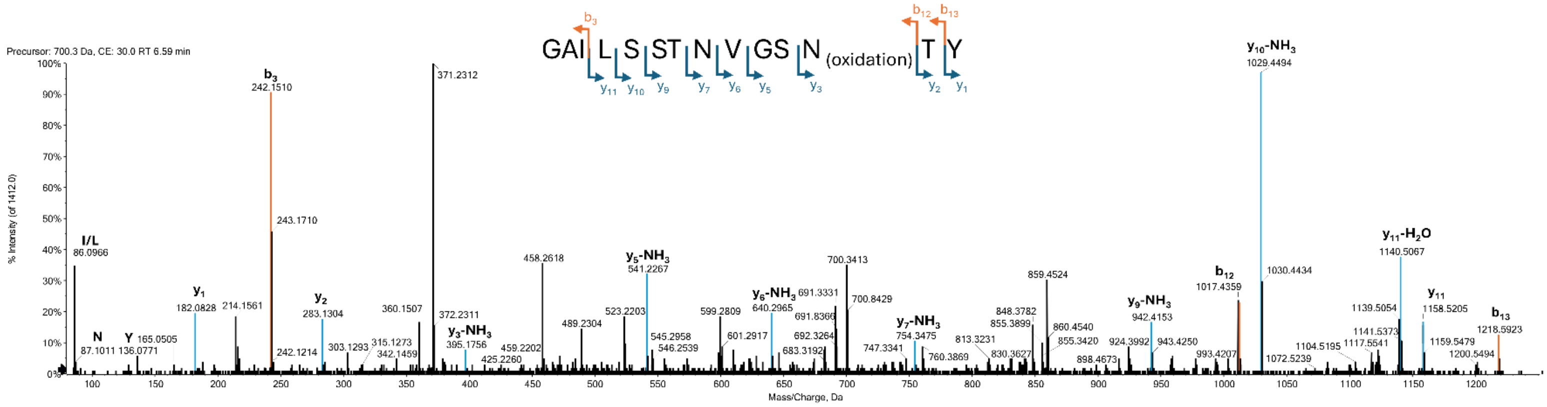

Precursor: 700.3 Da, CE: 30.0 RT 6.59 min
GAI L S ST N V GS N (oxidation) T Y
b3 b12 b13
y11 y10 y9 y7 y6 y5 y3 y2 y1
% Intensity (of 1412.0)
Mass/Charge, Da
I/L 86.0966
N 87.1011
Y 136.0771
165.0505
y1 182.0828
214.1561
b3 242.1510
242.1214
243.1710
y2 283.1304
303.1293
315.1273
342.1459
360.1507
371.2312
372.2311
y3-NH3 395.1756
425.2260
458.2618
459.2202
489.2304
523.2203
y5-NH3 541.2267
545.2958
546.2539
599.2809
601.2917
y6-NH3 640.2965
691.3331
691.8366
692.3264
683.3192
700.3413
700.8429
747.3341
y7-NH3 754.3475
760.3869
813.3231
830.3627
848.3782
855.3899
859.4524
860.4540
855.3420
898.4673
924.3992
y9-NH3 942.4153
943.4250
993.4207
b12 1017.4359
y10-NH3 1029.4494
1030.4434
1072.5239
1104.5195
1117.5541
1139.5054
1141.5373
y11-H2O 1140.5067
y11 1158.5205
1159.5479
1200.5494
b13 1218.5923

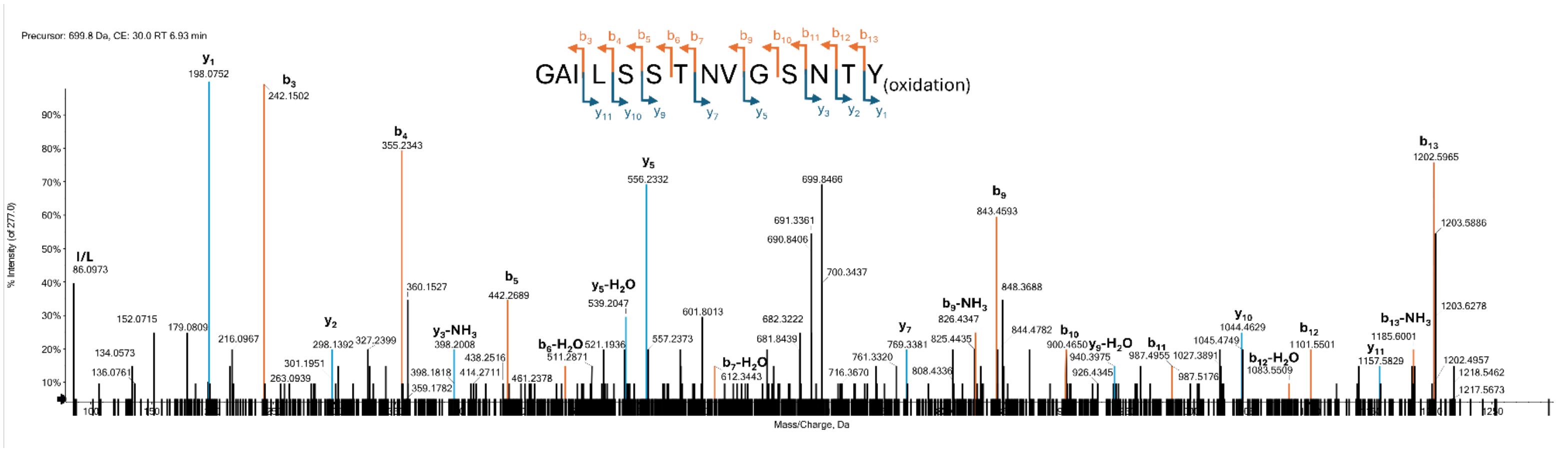

Figure S3. Identification of chymotryptic [H18A]IAPP oxidation sites by LC-MS/MS. Annotated high-resolution MS/MS spectra of (**A**) ^{1}KCNTATCATQRLANF^{15}, (**B**) ^{16}LVASSNNF^{23} and (**C**) ^{24}GAILSSTNVGSNTY^{37} peptides in their native and oxidized forms. Series of b- and y- ions are labeled in orange and blue, respectively. Precursor ions are highlighted in green and indicated above the spectra when detected. Spectra were acquired in CID mode (collision energy: 30 ± 10 V; cycle time: 1.05 s) with precursor charge states of z =+3 for [H18A]IAPP$_{(1-15)}$ and z =+2 for [H18A]IAPP$_{(16-23)}$ and [H18A]IAPP$_{(24-37)}$. Fragment assignments were made with a mass tolerance of ± 5 ppm.
